# Potential Natural Small Molecular Compounds for the Treatment of Chronic Obstructive Pulmonary Disease: An Overview

**DOI:** 10.3389/fphar.2022.821941

**Published:** 2022-03-24

**Authors:** Liu-Ying Li, Chuan-Tao Zhang, Feng-Ya Zhu, Gang Zheng, Yu-Fei Liu, Ke Liu, Chen-Hui Zhang, Hong Zhang

**Affiliations:** ^1^ Department of Heart Disease of Traditional Chinese Medicine, First People’s Hospital of Zigong City, Zigong, China; ^2^ Department of Respiratory Medicine, Hospital of Chengdu University of Traditional Chinese Medicine, Chengdu, China; ^3^ Department of Respiratory and Critical Care Medicine, First People’s Hospital of Zigong City, Zigong, China; ^4^ Department of Combine Traditional Chinese and Western Medicine, Hospital of Chengdu University of Traditional Chinese Medicine, Chengdu, China

**Keywords:** chronic obstructive pulmonary disease, natural compounds, flavonoids, polyphenol, alkaloid

## Abstract

Chronic obstructive pulmonary disease (COPD) is one of the major diseases threatening human life and health. According to the report released by the World Health Organization (WHO) in 2020, COPD has become the third leading cause of death in the world, featuring a sustainable growth of incidence rate as well as population age. The purpose of this review focuses on the advancement of bioactive natural compounds, such as baicalin, quercetin, resveratrol, and curcumin, which demonstrate promising therapeutic/interventional effects on CODP *in vitro* and *in vivo*. Information emphasizing on COPD was systematically collected from several authoritative internet databases including Web of Science, PubMed, Elsevier, Wiley Online Library, and Europe PMC, with a combination of keywords containing “COPD” and “natural small molecular compounds”. The new evidence indicated that these valuable molecules featured unique functions in the treatment of COPD through various biological processes such as anti-inflammatory, anti-oxidant, anti-apoptosis, and anti-airway fibrosis. Moreover, we found that the promising effects of these natural compounds on COPD were mainly achieved through JAK3/STAT3/NF-κB and MAPK inflammatory signaling pathways, Nrf2 oxidative stress signaling pathway, and TGF-β1/Smad 2/3 fibrosis signaling pathway, which referenced to multiple targets like TNF-α, IL-6, IL-8, TIMP-1, MMP, AKT, JAK3, IKK, PI3K, HO-1, MAPK, P38, ERK, etc. Current challenges and future directions in this promising field are also discussed at the end of this review. For the convenience of the readers, this review is divided into ten parts according to the structures of potential natural small molecular compounds. We hope that this review brings a quick look and provides some inspiration for the research of COPD.

## Introduction

Chronic obstructive pulmonary disease (COPD) is the third leading cause of mortality worldwide characterized by bronchitic and emphysematous components (Vogelmeier et al., 2020; Radicioni et al., 2021). The epidemiological survey of COPD shows that the prevalence of COPD in Spain has rapidly risen from 10.2 to 12.4%, and the proportion of men and women increases with age (Miravitlles et al., 2009). While there are about 175 million people around the world suffering from COPD, and the expenses for COPD-related treatment are as high as tens of billions of dollars every year. Only in the United States, the direct expenditure on COPD treatment in 2010 was $32 billion (Guarascio et al., 2013). It is generally believed that COPD is a series of pathophysiologic changes caused by inhaling pollutants (mainly cigarette smoke) or pathogens such as *Haemophilus influenzae*, *Moraxella catalhalis,* and *Streptococcus pneumoniae* (Leung et al., 2017; Szucs et al., 2019). The former can lead to airway inflammation by activating lung epithelium and inflammatory cells, while the latter can trigger pathogen-associated molecular patterns through pattern recognition receptors expressed on epithelial cells and innate immune cells, and activate nuclear factors κB (NF-κB), mitogen activated protein kinase (MAPK), phosphatidylinositol-3-kinase (PI3K), and IFN regulator signal, which lead to the production of pro-inflammatory mediators such as cytokines and chemokines, and cause a sustained harmful immune response (Sethi et al., 2006; Hallstrand et al., 2014). Subsequently, these persistent immune and inflammatory reactions will gradually introduce airway structural changes, and cause obstruction and respiratory symptoms (Rovina et al., 2013).

**FIGURE 1:**
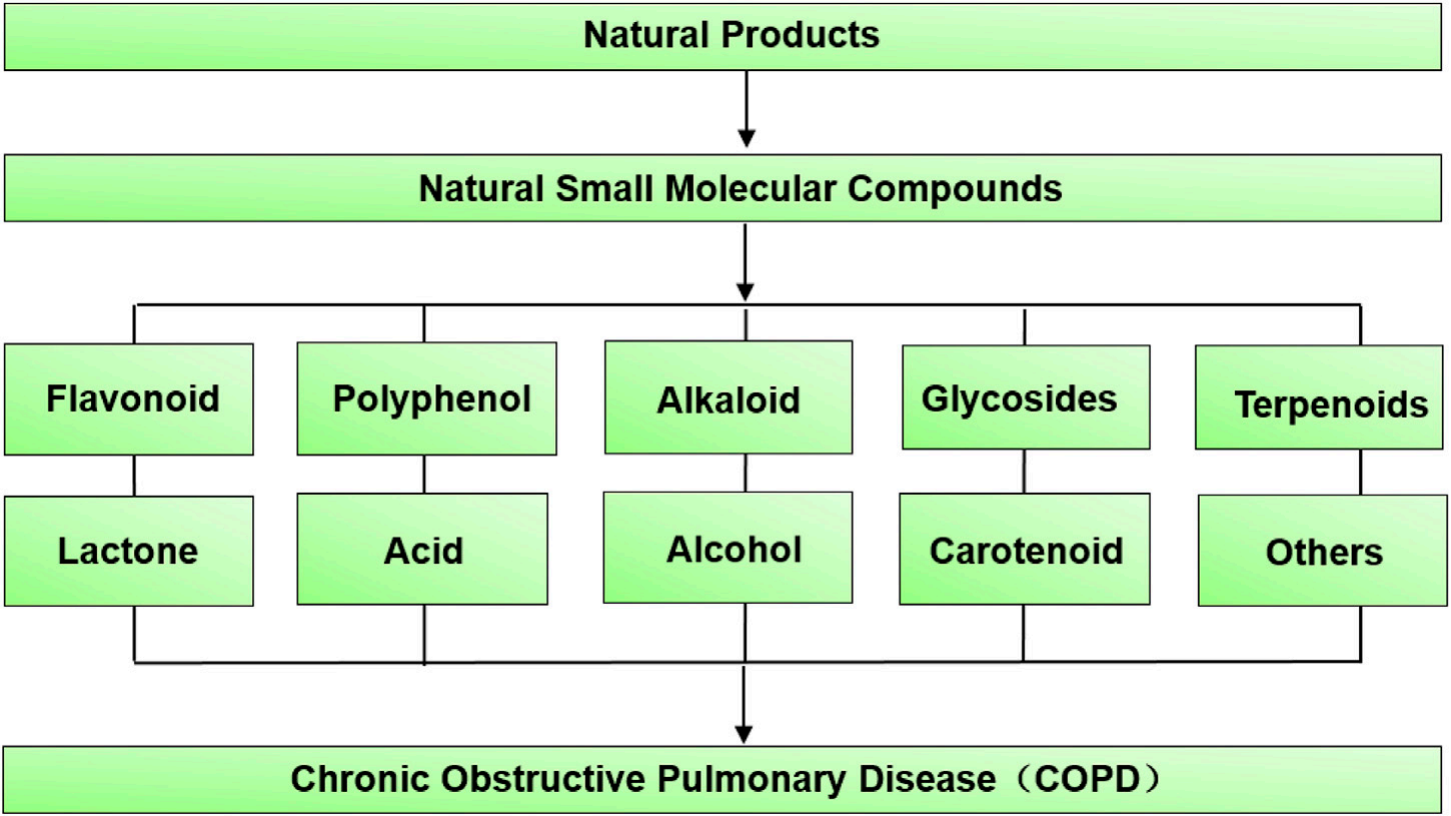
The 10 kinds of natural small molecular compounds that have an effect on COPD.

**FIGURE 2:**
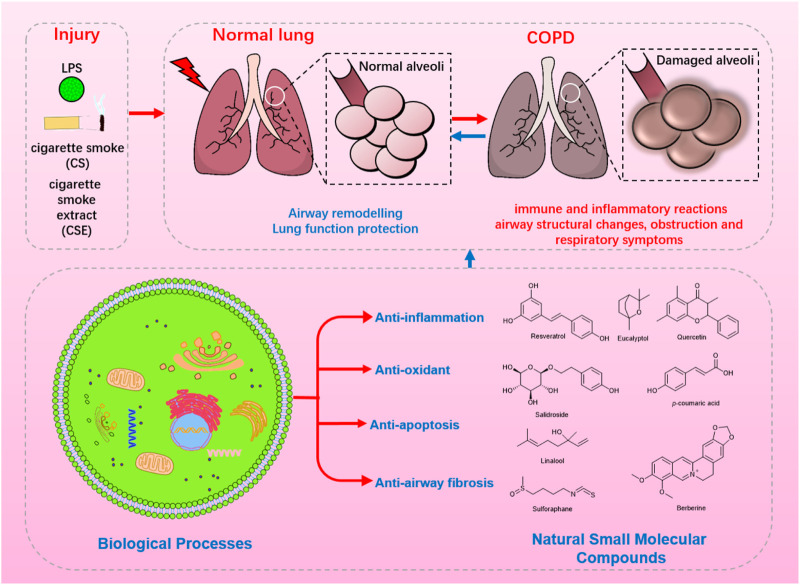
The biological processes and mechanisms of natural small molecular compounds in the treatment of COPD.

**FIGURE 3:**
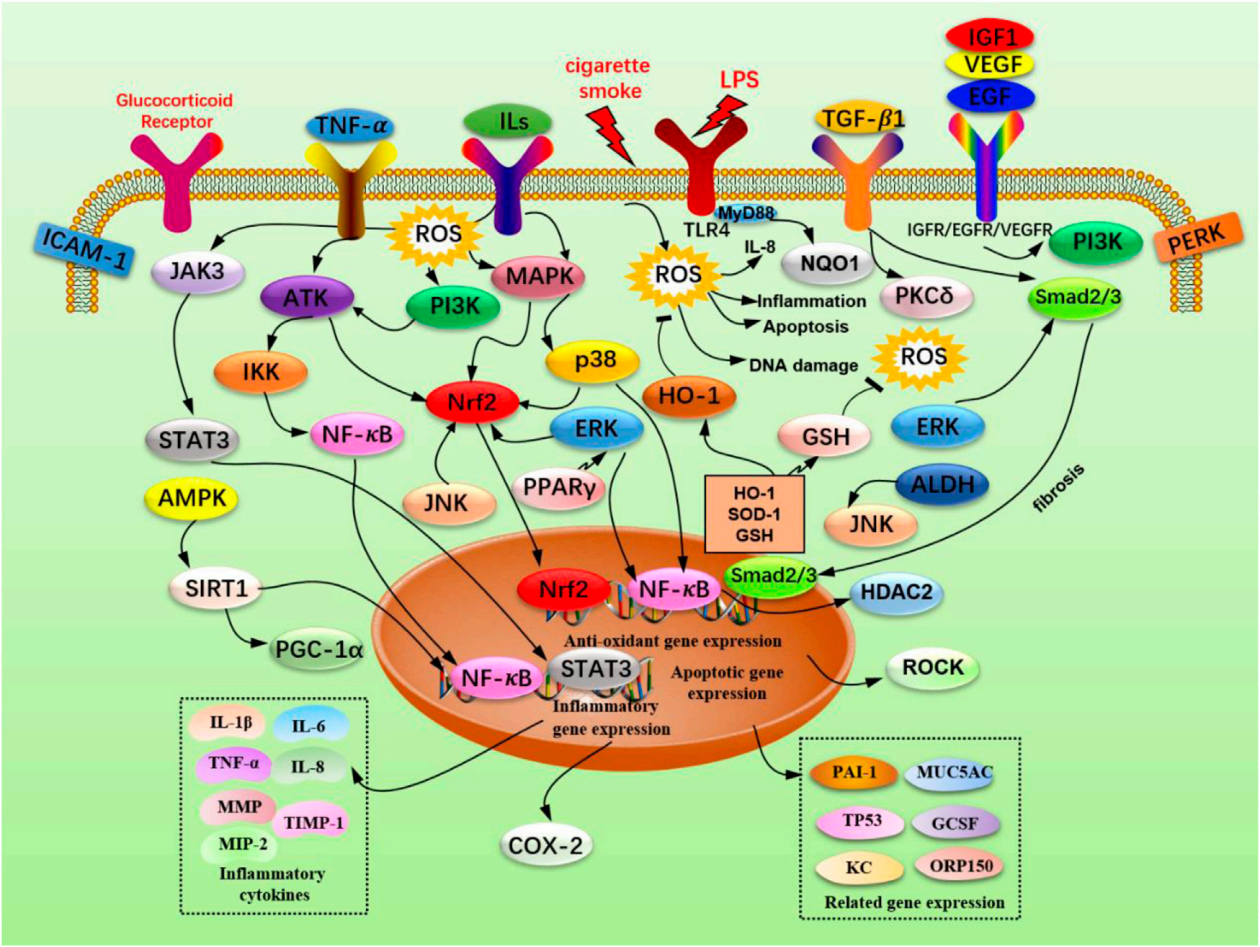
The signaling pathways of natural small molecular compounds in the treatment of COPD.

An increasing number of evidences indicate that inflammation serves as the turning point of vascular reconstitution in COPD, which is associated with the untimely activation of epithelial cells and innate immune cells (such as neutrophils, eosinophils, and macrophages) with inflammatory mediators (i.e. inflammatory peptides, lipid mediators, growth factors, reactive oxygen and nitrogen species, chemokines cytokines, and cellular proteases) (Barnes, 2016; Kuźnar-Kamińska et al., 2018; Capron et al., 2019). Recent studies have shown that numerous natural compounds possess obvious therapeutic effects on the symptoms of COPD model animals (Figure 1). For example, not only can quercetin, a

representation of flavonoid, significantly reduce pulmonary oxidative stress, inflammation, and mucus production in COPD model animals, but also improve corticosteroid resistance by promoting AMPK activation and Nrf2 expression (Ganesan et al., 2010; Braun et al., 2011; Mitani et al., 2017). The polyphenol compound curcumin enables the promotion of airway inflammation and airway remodeling in COPD model animals by regulating the NF-κB signaling pathway (Yuan et al., 2018), and improves skeletal muscle dysfunction by up-regulating the PGC-1α/SIRT3 pathway (Zhang et al., 2017). In addition, curcumin demonstrates an inhibition on the expression of pro-inflammatory genes and the level of chemokines, and regulations towards corticosteroid resistance (Gan et al., 2016). Given these multiple effects on improving COPD symptoms, a prospective review regarding the advancements of potential natural small molecular compounds is highly needed. However, to the best of our knowledge, less attention has been paid in this promising field despite scattered summaries in several reviews (Goncalves and Romeiro, 2019). The main purpose of this review concentrates on summarizing the latest and representative information on therapeutic/interventional effects of reported natural compounds on COPD in recent years (Figure 2), to excavate the potential of these bioactive molecules and furnish basic information for research in the future, as well as provide a useful supplement to reviews related to COPD (Figure 3).

## Flavonoid

**FIGURE 4:**
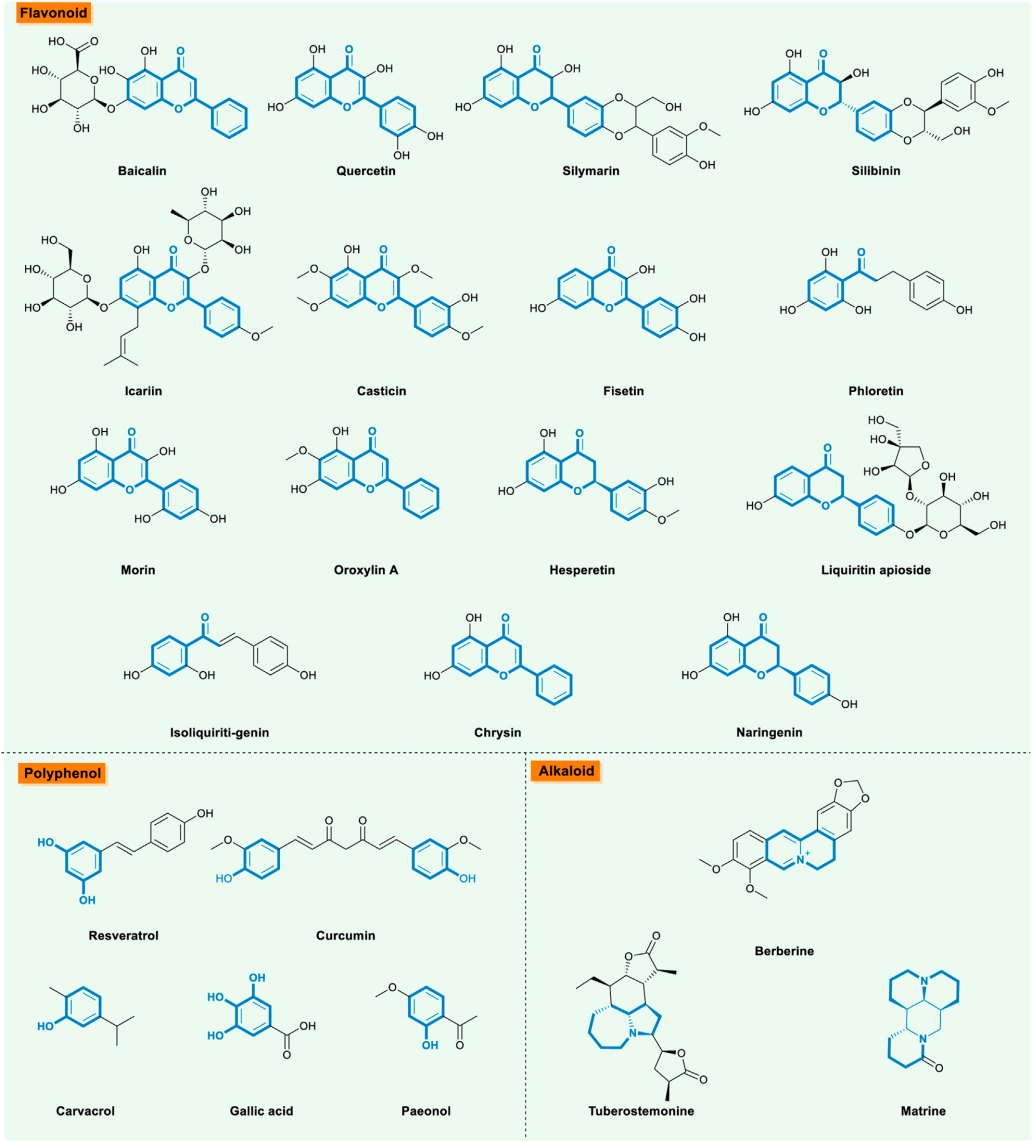
The bioactive natural compounds from flavonoid, polyphenol, and alkaloid.

Flavonoids possess a variety of biological properties such as anti-inflammatory, anti-apoptosis, and anti-oxidant properties to improve COPD symptoms (Table 1; Figure 4). Baicalin is a flavonoid compound isolated from the root of *Scutellaria baicalensis Georgi*, possessing multiple biological activities, such as anti-inflammatory and anti-oxidant properties. To clarify the effects of baicalin on COPD, the mice and cell models were established by using cigarette smoke (CS) and cigarette smoke extract (CSE), respectively. Results showed that baicalin could regulate pro-infammatory and anti-infammatory balance and exert great lung function protection on COPD (Lixuan et al., 2010; Li et al., 2012; Wang et al., 2018a; Hao et al., 2021; Zhang et al., 2021). The anti-inflammatory effect was likely achieved via inhibiting the nuclear factor-kappa B(NF-κB) activation (Lixuan et al., 2010), up-regulating histone deacetylase 2(HDAC2) protein expression, along with inhibiting HDAC2 phosphorylation (enhancing HDAC2 activity) (Li et al., 2012), and modulating HDAC2/NF-κB/PAI-1 signaling pathways (Zhang et al., 2021).

**TABLE 1:** The effects of flavonoid on COPD.

Flavonoids	Sources	Models	Effects	Dose	Application	Ref
Baicalin	Scutellaria baicalensis Georgi	*In vivo*: COPD mice model was established by cigarette smoke (CS) exposure	Inhibition of the NF-kB pathway	20–80 mg/kg	*In vivo*	Lixuan et al. (2010)
		*In vitro*: cell model was established by using cigarette smoke extract (CSE) to stimulate type-II pneumocytes		5–20 μM	*In vitro*	
		CS-induced inflammatory models in mice; CSE-induced inflammatory models in A549 cells	Modulating HDAC2/anti-inflammatory	25–100 mg/kg	*In vivo*	Li et al. (2012)
				10–100 μM	*In vitro*	
		CS-induced rat model of COPD	Anti-infammatory/anti-airway remodeling/antioxidant	40–160 mg/kg	*In vivo*	Wang et al. (2018a)
		CS/CSE-induced airway inflammation in mice or human bronchial epithelial (HBE) cells	Anti-infammatory	40–160 mg/kg	*In vivo*	Zhang et al. (2021)
				10–40 μM	*In vitro*	
		CSE-induced MLE-12 cells; CS-induced COPD mice model	Regulation of HSP72-mediated JNK pathway	25–100 mg/kg	*In vivo*	Hao et al. (2021)
				5–20 μmol/L	*In vitro*	
Quercetin	Polygoni avicularis herba	CSE-induced muman monocytic U937 cells and peripheral blood mononuclear cells (PBMC) collected from patients with COPD	Increased AMPK activation and Nrf2 expression, and restored corticosteroid resistance	10 μM	*In vitro*	Mitani et al. (2017)
		CSE-induced mice model/human airway epithelial NCI-H292 cells	Inhibiting the NF-κB pathway and EGFR phosphorylation	25–50 mg/kg	*In vivo*	Yang et al. (2012)
				5–20 μM	*In vitro*	
		Primary human osteoblasts exposed to cigarette smoke medium (CSM)	Activation of the anti-oxidative enzymes HO-1 and SOD-1	25–100 μM	*In vitro*	Braun et al. (2011)
		Elastase/lipopolysaccharide (LPS)-exposed mice	Negatively regulating MMP expression	10 mg/kg	*In vivo*	Ganesan et al., 2010
		Rhinovirus-infected mice with COPD phenotype	Preventing progression of lung disease in COPD	0.1% quercetin containing diet	*In vivo*	Farazuddin et al. (2018)
Silymarin	Silybum marianum	CS-induced mice mode	Suppression of inflammation and oxidative stress by inhibiting the ERK/p38 MAPK pathway	25–50 mg/kg	*In vivo*	Li et al. (2015)
		CSE-induced human bronchial epithelial cell line (BEAS-2B) model	Inhibition of autophagy and the ERK/p38 MAPK pathway	10–40 μM	*In vitro*	Li et al. (2016a)
Silibinin	Silybum marianum	CS and LPS exposure-induced mice model	Inhibited the pulmonary fibrosis induced by CS via suppression of TGF-β1/Smad 2/3 signaling	10–20 mg/kg	*In vivo*	Ko et al. (2017)
		CS-/LPS-induced COPD model mice; CS condensate-stimulated H292 cells	Inhibition in ERK phosphorylation	20–40 mg/kg	*In vivo*	Park et al. (2016)
				6.25–50 μg/ml	*In vitro*	
Icariin	Epimedium	CSE-exposed BEAS-2B cells model	Reversing Glucocorticoids (GC)resistance	20–80 µM	*In vitro*	Hu et al. (2020)
		CS-induced lung inflammation using BALB/c mice; CSE-exposed A549 epithelial cells	Ameliorated inflammation by suppressing NF-kB activation and modulating glucocorticoid receptor (GR) protein expression	25–100 mg/kg	*In vivo*	Li et al. (2014)
				10–100 µM	*In vitro*	
Casticin	Vitex rotundifolia and Vitex agnus-castus	CS-induced C57BL/6 mice model	Inhibition of inflammatory cytokines and chemokines	1–10 mg/kg	*In vivo*	Lee et al. (2015)
		CS-exposed mice	Attenuated oxidative Stress and inflammation via inhibition of NF-ĸB	10–30 mg/kg	*In vivo*	Li et al. (2020)
Fisetin	Gleditsiae spina	Human airway epithelial cells	Inhibiting the TNF-α/NF-κB signaling pathway	2.5–10 μM	*In vitro*	Lee et al. (2018a)
		CS-exposed mice	Up-regulation of Nrf2 expression	50 mg/kg	*In vivo*	Hussain et al. (2019)
Phloretin	Crotonis fructus; Rubi fructus	CS-induced mice model; CSE-induced NCI-H292 cells model	Inhibition of epidermal growth factor receptor (EGFR)/MAPK signaling pathways	10–20 mg/kg	*In vivo*	Wang et al. (2018b)
				1–10 μM	*In vitro*	
Morin	Cudrania tricuspidata	CS-induced mice model	Anti-inflammation via inhibiting the P13K/ATK/NF-κB signaling pathway	10–40 mg/kg	*In vivo*	Cai et al. (2018)
Oroxylin A	Scutellaria baicalensis Georgi	CS-stimulated BEAS-2B cells and RAW264.7 cells; CS-induced mice	Activating the Nrf2 signaling pathway	15–60 mg/kg	*In vivo*	Li et al. (2016b)
				50–150 μM	*In vitro*	
Hesperetin	Citrus reticulata	CSE-induced mice model	Regulation of SIRT1/PGC-1α/NFκ-B signaling axis	25–50 mg/kg	*In vivo*	Wang et al. (2020)
		CS- and urethane-induced lung cancer with COPD in mice	Preventing COPD progression to lung cancer	25–100 mg/kg	*In vivo*	Zhou et al. (2021)
Liquiritin apioside	Glycyrrhiza uralensis	CSE-induced cell injury in the A549 lung epithelial cell; CS-induced mice inflammation model	Inhibiting TGF-β and TNF-α expression and increasing levels of GSH	3–30 mg/kg	*In vivo*	Guan et al. (2012)
				108–106 M	*In vitro*	
Isoliquiriti-genin	liquorice	CS-induced mice model	Regulating the Nrf2 and NF-κB signaling pathways	10–30 mg/kg	*In vivo*	Yu et al. (2018a)
Chrysin	Flowers	CS-induced airway inflammation in mice	Inhibition of ERK and p38 phosphorylation	10–20 mg/kg	*In vivo*	Shen et al. (2015)
Naringenin	Amacardi-um occidentale L	CS-induced mice model; CSE-exposed A549 cells	Suppression of NF-κB	20–80 mg/kg	*In vivo*	Liu et al. (2018)
					*In vitro*	

As a flavonoid abundant in fruits and vegetables, quercetin has attracted much attention for its beneficial health effects including anti-oxidant and anti-inflammation activity. It was found that quercetin successfully reduced oxidative stress, lung inflammation, and mucus production via negating MMP expression in elastase/LPS-exposed mice (Ganesan et al., 2010), or via inhibiting the NF-κB pathway and EGFR phosphorylation both in the CS/CSE-induced mice model and NCI-H292 cell model (Yang et al., 2012). Smokers frequently suffer from impaired fracture healing often due to poor bone quality and stability induced by increasing formation of reactive oxygen species (ROS). One research found that quercetin could protect primary human osteoblasts from the toxic effects of smoking through activation of the anti-oxidative enzymes HO-1 and SOD-1 (Braun et al., 2011). Besides, acute exacerbations are the major cause of morbidity and mortality in patients with COPD, Mohammad Farazuddin et al. disclosed that quercetin effectively mitigated rhinovirus-induced progression of lung disease on COPD mice models (Farazuddin et al., 2018). To remove a major barrier known as corticosteroid resistance for the effective treatment of COPD, quercetin also provided access to restore corticosteroid sensitivity in cells from patients with COPD via the mechanism of increasing AMPK activation and Nrf2 expression (Mitani et al., 2017).

Separated from the milk thistle (*Silybum marianum*), silymarin attenuated inflammation and oxidative stress induced by CS/CSE on mice and in the BEAS-2B cell (human bronchial epithelial cells). The anti-inflammatory and anti-oxidant effects of silymarin might be related to the inhibition of autophagy and ERK/p38 MAPK pathway (Li et al., 2015; Li D. et al., 2016). Silibinin, an active constitute of silymarin, could markedly reduce the production of fibrotic mediators in CS + LPS-exposed mice via suppression of TGF-β1/Smad 2/3 signaling (Ko et al., 2017), as well as clearly decrease the pro-inflammatory mediators and airway mucus production expression in CS condensate-stimulated H292 cells and COPD mice model via the inhibition in ERK phosphorylation (Park et al., 2016). Among dihydroflavones, Naringenin, hesperetin, and liquiritin apioside (LA) also exhibited positive effects on COPD, among which hesperetin could not only effectively alleviate inflammation and oxidative stress responses in CES-induced COPD mice by virtue of NAD-dependent protein deacetylase sirtuin-1(SIRT1)/PGC-1α/NF-κB signaling axis (Wang et al., 2020), but also suppress the protein expression of AKT1, IL6, VEGFA, and MMP9 and up-regulate TP53 to reduce the risk of COPD progressing to lung cancer (Zhou et al., 2021). Besides, LA offered protection to lung epithelial cell from CS-induced injuries by inhibiting the transforming growth factor-*β* (TGF-β) and tumor necrosis factor-α (TNF-α) expression and increasing anti-oxidative levels of glutathione (GSH) (Guan et al., 2012). Notably, naringenin smoothly attenuated inflammation in COPD on CS-induced mice models via suppressing NF-κB pathway (Liu et al., 2018).

As a major constituent of flavonoids isolated from the herb *Epimedium*, icariin exerted a therapeutic effect in numerous chronic inflammatory diseases. However, COPD tends to be glucocorticoid (GC) resistant, and Lingli Hu et al. noted that icariin was able to decrease CSE-induced inflammation, airway remodeling, and ROS production by mitigating GC resistance in CSE-induced BEAS-2B cells models (Hu et al., 2020). Besides, icariin owned anti-inflammatory effects on CS-induced inflammatory models, which was possibly achieved by suppressing NF-κB activation and modulating the glucocorticoid receptor (GR) protein expression (Li et al., 2014). Except for icariin suppressing NF-κB activation, casticin, which was a poly-methylflavone obtained from *Vitex* species such as *Vitex rotundifolia* and *Vitex agnus-castu*s, was found possessing significant effects on attenuating oxidative stress and lung inflammation induced by CS (Lee et al., 2015), which was related to the inhibition of NF-ĸB pathway (Li et al., 2020). Apart from the extractions from herbs, the natural flavonoid fisetin (3,7,3′,4′-tetrahydroxyflavone) demonstrated its abilities on effectively alleviating lung oxidative stress and inflammation induced by the powerful pro-oxidant CS through the incremental expression of Nrf2 as well as its downstream target anti-oxidant gene (Hussain et al., 2019). Furthermore, Seoghyun Lee et al. found that fisetin acted as a good drug candidate for improving the lung function of patients with COPD by suppressing the TNF-α/NF-κB signaling cascade (Lee S. et al., 2018). Additionally, the valuable chalcone phloretin existing in C*rotonis fructus* and *Rubi fructus* featured diverse biologic properties. Hao Wang et al. reported that phloretin-based pre-treatment remarkably blocked mucins secretion, inflammatory cytokine release, and inflammatory cell infiltration on CS-induced mice models, as well as an interruption of CSE-induced expression of MUC5AC and IL-1β in NCI-H292 bronchial epithelial cells. Those previously mentioned protections were possibly achieved by attenuating the functions of P38, ERK and EGFR *in vivo* and *in vitro* (Wang et al., 2018b).

Despite the similarity of morin, oroxylin A and chrysin on structures, the three natural products provided positive effects on COPD through different mechanisms. Briefly, morin (3,5,7,2′,4′-pentahydroxyflavone), a major component of a traditional medicinal herb *Cudrania tricuspidata*, demonstrated protective effects on CS-induced lung inflammation probably by blocking P13K/ATK/NF-κB signaling pathway (Cai et al., 2018); Known as a natural flavonoid extracted from the traditional herb *Scutellaria baicalensis Georgi*, oroxylin A attenuated CS-induced lung histopathologic changes, expression of cytokines TNF-α, IL-1β in a mice model with a dose-dependent manner, as well as significantly up-regulated Nrf2 expression in CSE-stimulated cells (Li J. et al., 2016). Furthermore, as a naturally-occurring flavone commonly found in flowers, chrysin effectively inhibited CSE-induced airway inflammation in mice through inhibition of ERK and p38 phosphorylation (Shen et al., 2015). Beyond these molecules mentioned previously, as a variant of flavonoid, isoliquiriti-genin (ILG) derived from the root of liquorice was reported to antagonize COPD on CS-induced mice model by suppressing inflammatory and oxidative stress through up-regulating the expression of Nrf2 and down-regulating the expression of NF-κB signaling pathways (Yu D. et al., 2018).

## Polyphenol

Polyphenol belongs to a group of chemical substances in plants featuring multiple phenol groups (Table 2; Figure 4). Resveratrol (3,4′,5-trihydroxystilbene; RESV), a natural polyphenol phytoalexin identified from a variety of plant species, exhibited a protective effect against CSE-induced apoptosis in cells (Zhang L. et al., 2015; Song et al., 2017; Zong et al., 2021). The anti-apoptotic effect may be exerted through the activation of a pathway involving SIRT1 and ORP150 in CSE-induced HBEpC cell (Zhang L. et al., 2015), and activation of Notch1 signaling mediated autophagy in CSE-induced HUVECs models (Zong et al., 2021), or via up-regulating mitofusin 2 (MFN2) in a CSE-induced HBEpC cell (Song et al., 2017). Recently, studies have found that resveratrol could protect against oxidative damage and pulmonary inflammation on the COPD mice model (Liu et al., 2014a; Wang et al., 2017), where the mechanism might be related with decreasing NF-κB activity and elevated HO-1 expression, and activating the SIRT1/PGC-1α signaling pathways (Wang et al., 2017). Alongside the functions mentioned previously, resveratrol could not only effectively attenuate the release of inflammatory cytokines from human bronchial smooth muscle cells (HASMCs) in COPD (Knobloch et al., 2010; Knobloch et al., 2014), but also inhibit the NF-κB, TNF-α, and MMP-9-associated pathways, simultaneously slowing the dysfunction of dendritic cells (DCs) in patients with COPD (Wang et al., 2015; Liu et al., 2016). These findings proved that resveratrol was able to ameliorate cardiac oxidation stress and apoptosis and increase the expression of SIRT1, as well as attenuate left ventricular remodeling, while these factors might assist the left ventricular impairment process in old mice with COPD induced by CS and LPS exposure. (Hu et al., 2013). Overall, resveratrol prophylaxis by inhalation is a potential approach for slowing down ageing-related deterioration of the lung function and structure in prematurely ageing telomerase null (terc−/−) mice, which could be developed as a potentially novel approach to maintaining lung health, prior to the irreversible onset of ageing-related structural and functional decline in the lungs (Navarro et al., 2017).

**TABLE 2:** The effects of polyphenol on COPD.

Polyphenol		Sources	Models	Effects	Dose	Application	Ref
Resveratrol	Various plants, nuts and fruits		CSE-induced HBE cell model	Anti-apoptotic effect through the activation SIRT1 and ORP150	20 μmol/L	*In vitro*	Zhang et al. (2015b)
			CSE-induced Human umbilical vein endothelial cells (HUVECs) model	Anti-apoptosis	40 μM	*In vitro*	Zong et al. (2021)
			CSE-induced HBE cells model	Reduced apoptosis	20 μM	*In vitro*	Song et al. (2017)
			CS-induced mice model	Decreased NF-κB activity and the elevated HO-1 expression and activity	1–3 mg/kg	*In vivo*	Liu et al. (2014a)
			CS- and LPS-induced lung inflammation in a mouse model of COPD	Activating SIRT1/PGC-1α signaling pathways	50 mg/kg	*In vivo*	Wang et al. (2017)
			Human bronchial smooth muscle cells (HASMCs) exposed to lipoteichoic acid (LTA)	Anti-inflammation	10-6-10-4M	*In vitro*	Knobloch et al. (2014)
			Lymphocytes isolated from patients with COPD	Inhibited the translocation of NF κB, and decreased TNF α	12.5 μmol/l	*In vitro*	Liu et al. (2016)
			Human airway smooth muscle cells	Anti-inflammatory	10-7-10–3 M	*In vitro*	Knobloch et al. (2010)
			Dendritic cells (DCs) from COPD patients	Inhibited dysfunction of dendritic cells (DCs)	10 μmol/ml	*In vitro*	Wang et al. (2015)
			Old mice with COPD induced by CS exposure and LPS instillation	Attenuated left ventricular remodeling	25 mg/kg	*In vivo*	Hu et al. (2013)
			Prematurely ageing telomerase null (terc−/−) mice	Slowed ageing-related degenerative changes in mouse lungs	1 mg/kg	*In vivo*	Navarro et al. (2017)
Curcumin	Curcuma longa		In mice model of COPD-like airway inflammation induced by non-typeable haemophilus influenzae exposure (NTHi)	Inhibition of inflammation and lung cancer progression	0.2–2%	*In vivo*	Moghaddam et al. (2009)
			LPS- and CS-induced COPD murine models; LPS-stimulated BEAS-2B cells	Inhibiting NF-κB Signaling and COX-2	100–200 mg/kg	*In vivo*	Yuan et al. (2018)
					0.1–10 μmol/L	*In vitro*	
			CSE-treated BEAS-2B cells; CS-induced COPD mice models	Modulating the PPARγ-NF-κB signaling pathway	100 mg/kg	*In vivo*	Li et al. (2019)
					2.5–7.5 mΜ	*In vitro*	
			Patients with mild COPD	Reduced serum atherosclerotic low-density lipoprotein levels in patients with mild COPD	180 mg	*In vivo*	Funamoto et al. (2016)
			Mice model of COPD established by CSE combined with lipopolysaccharide	Up-regulation of PGC-1α/SIRT3 signaling pathway	100 mg/kg	*In vivo*	Zhang et al. (2017)
			*In vitro* model of CSE-induced inflammation using human monocytic cell line (U937)	Restored corticosteroid function in monocytes exposed to oxidants by maintaining HDAC2	1–10,000 nM	*In vitro*	Meja et al. (2008)
			CSE-induced mice model with COPD	Modulating HDAC2 expression and its effect on histone modification	100 μM	*In vitro*	Gan et al. (2016)
Carvacrol	Zataria multiflora Boiss		Elastase-induced emphysema mice	Anti-inflammatory via suppression of NF-κB	20 mg/kg	*In vivo*	Games et al. (2016)
			Guinea pigs model of COPD induced by CSE	Attenuated systemic inflammation	60–240 μg/ml	*In vitro*	Mahtaj et al. (2015)
			Guinea pigs model of COPD exposed to CS	Prevention of tracheal responsiveness and emphysema	60–240 μg/ml	*In vitro*	Gholami Mahtaj et al. (2015)
			Guinea pigs model of COPD exposed to CS	Against lung inflammation and oxidative stress	60–240 μg/ml	*In vivo*	Boskabady and Gholami Mahtaj, (2015)
Gallic acid	Rheum palmatum L		Elastase (ET-) + LPS- induced COPD exacerbation like condition in mice model	Prevented the activation of NF κB and elevated the expression of Nrf2	200 mg/kg	*In vivo*	Singla et al. (2021)
			ET- and CS-induced mice model	Suppressed phosphorylation of p65NF-κB and IκBα along with down-regulation of IL-1β/TNF-α/KC/MIP-2/GCSF genes	200 mg/kg	*In vivo*	Singla et al. (2020)
Paeonol	Paeonia suffruticosa		CS-induced mice model/CSE-induced HBE cell model	Inhibition of the MAPKs/NF-κB signaling	10 mg/kg	*In vivo*	Liu et al. (2014b)
					0.05–0.4 mM	*In vitro*	

Curcumin [(1E,6E)-1,7-bis(4-hydroxy-3-methoxyphenyl)hepta-1,6-diene-3,5-dione] is a naturally occurring polyphenolic phytochemical isolated from the rhizome of the medicinal plant *Curcuma longa.* Dietary administration of curcumin effectively suppressed NTHi-induced COPD-like airway inflammation and lung cancer progression in mice (Moghaddam et al., 2009). Curcumin could also attenuate CS-induced inflammation both *in vivo* and *in vitro* by modulating the PPARγ-NF-κB signaling pathway (Li et al., 2019), along with attenuating airway inflammation and remodeling by blocking NF-κB and COX-2 signaling on CS-induced COPD mice (Yuan et al., 2018). Theracurmin^®^, a highly absorptive curcumin, with improved bioavailability using a drug delivery system, reduced levels of the atherosclerotic α1-antitrypsin-low-density lipoprotein (AT-LDL) complex. This result suggested that curcumin was beneficial to prevent the development of vascular events in patients with COPD (Funamoto et al., 2016). Among the multiple symptoms induced by COPD, skeletal muscle

dysfunction is one of the most extrapulmonary symptoms in COPD patients, where mitochondria manifestation plays an important role in the duration. Therefore, protecting mitochondria from injury is crucial to prophylaxis skeletal muscle dysfunction during the progression of COPD. Under this context, Ming Zhang et al. found that curcumin smoothly attenuated skeletal muscle mitochondrial impairment in COPD mice via up-regulating the PGC-1α/SIRT3 signaling pathway (Zhang et al., 2017). In addition, recent studies have suggested that histone modification showed a positive impact on various aspects associated with the progression of COPD where histone deacetylase 2 (HDAC2) could suppress proinflammatory gene expression through deacetylation of core histones. Thus, Lixing Gan et al. investigated the functions variation of histone modification via a combination with the expression of chemokines in type-II alveolar epithelial cells (AEC II) and HDAC2 caused by curcumin on a mice model with COPD induced by CS, and the results indicated that curcumin might inhibit chemokines and rebuild corticosteroid resistance in COPD through modulating HDAC2 expression, as well as show influence on histone modification (Gan et al., 2016). Similarly, another study found that curcumin could restore CS-impaired HDAC2 activity and corticosteroid efficacy in monocytes (Meja et al., 2008). All in all, curcumin showed potential to reverse corticosteroid resistance, which is commonly observed in patients with COPD.

Carvacrol, C_6_H_3_CH_3_(OH) (C_3_H_7_) as a constituent of *Zataria multiflflora Boiss*, was reported to own preventive therapeutic potential on lung infection and oxidative stress on CS-induced guinea pig models with COPD, which was comparable to or more potent than the effect of dexamethasone at used concentrations (Boskabady and Gholami Mahtaj, 2015; Gholami Mahtaj et al., 2015; Mahtaj et al., 2015). Subsequently, Ellen Games et al. found that carvacrol could protect mice against elastase-induced emphysema through a suppression of the NF-κB pathway (Games et al., 2016). Like other naturally occurring phenolic compounds, gallic acid is known to possess anti-oxidant/anti-inflammatory activities. Researchers revealed that the gallic acid protected against COPD exacerbation manifestations through inversing modulation of redox sensitive transcription factors-NF-κB and Nrf2 (Singla et al., 2021). Meanwhile, gallic acid ameliorated elastase (ET)-induced inflammation and emphysema by the restoration of redox imbalance and inhibition of NF-κB activation (Singla et al., 2020). As the representative of phenolic, paeonol existing in the Chinese herb *Paeonia suffruticosa* has been identified with the optimistic effects on alleviating oxidative stress and lung inflammation on CS-induced mice models. In addition, paeonol could also suppress CSE-induced IL-8 and ROS in human bronchial epithelial cells (HBECs) via inhibition of the MAPKs/NF- *k*B signaling (Liu et al., 2014b).

## Alkaloid

Alkaloid, a class of naturally occurring organic nitrogen-containing bases, participates in diverse physiological functions of the human body (Table 3; Figure 4). As for COPD discussed in this review, berberine, as a protoberberine alkaloid, could effectively attenuate CS-induced lung inflammation in mice (Lin et al., 2013; Xu et al., 2015; Wang et al., 2019). Studies further confirmed that the anti-inflammation effect of berberine were associated with the suppression CS-induced NF-κB activation (Lin et al., 2013), inhibition of TGF-β1/Smads signaling (Wang et al., 2019), or inhibition of ERK and P38 pathway (Xu et al., 2015). Besides, as an alkaloid-type phytochemical from *Stemona tuberosa,* tuberostemonine (TS) attenuated CS-induced lung inflammation and decreased alveoli size in lung tissue through the inhibition of the infiltration of inflammatory cells by decreasing the chemokine expression related to lung inflammation (Jung et al., 2016a; Jung et al., 2016b). Apart from previously mentioned alkaloids, matrine, an alkaloid compound existed in *Sophora flavescens* Ait (Kushen) with a useful bioactivity of anti-inflammatory effect, Xuhua Yu et al. disclosed it could reduce CS-induced neutrophilic inflammation by inducing neutrophil apoptosis (Yu X. et al., 2019).

**TABLE 3:** The effects of alkaloids on COPD.

Alkaloid		Sources	Models	Effects	Dose	Application	Ref
Berberine	Coptidis Rhizoma		CS-induced mice model	Suppressed CS-induced NF-κB activation	50 mg/kg	*In vivo*	Lin et al. (2013)
			CSE-induced airway inflammation in mice	Inhibition of TGF-β1/Smads signaling	25 mg/kg	*In vivo*	Wang et al. (2019)
			Mice exposed to CS	Inhibition of ERK and P38 pathway	5–10 mg/kg	*In vivo*	Xu et al. (2015)
Tuberostemonine	Stemona tuberosa		CS-induced lung inflammation in mice	Suppressed inflammation	1–10 mg/kg	*In vivo*	Jung et al. (2016b)
			CS-induced mice model	Suppressed inflammation	1–10 mg/kg	*In vivo*	Jung et al. (2016a)
Matrine	Sophora flavescens Ait		CS-induced mice model	Inducing neutrophil apoptosis	100 mg/kg	*In vivo*	Yu et al. (2019b)

## Glycosides

**FIGURE 5:**
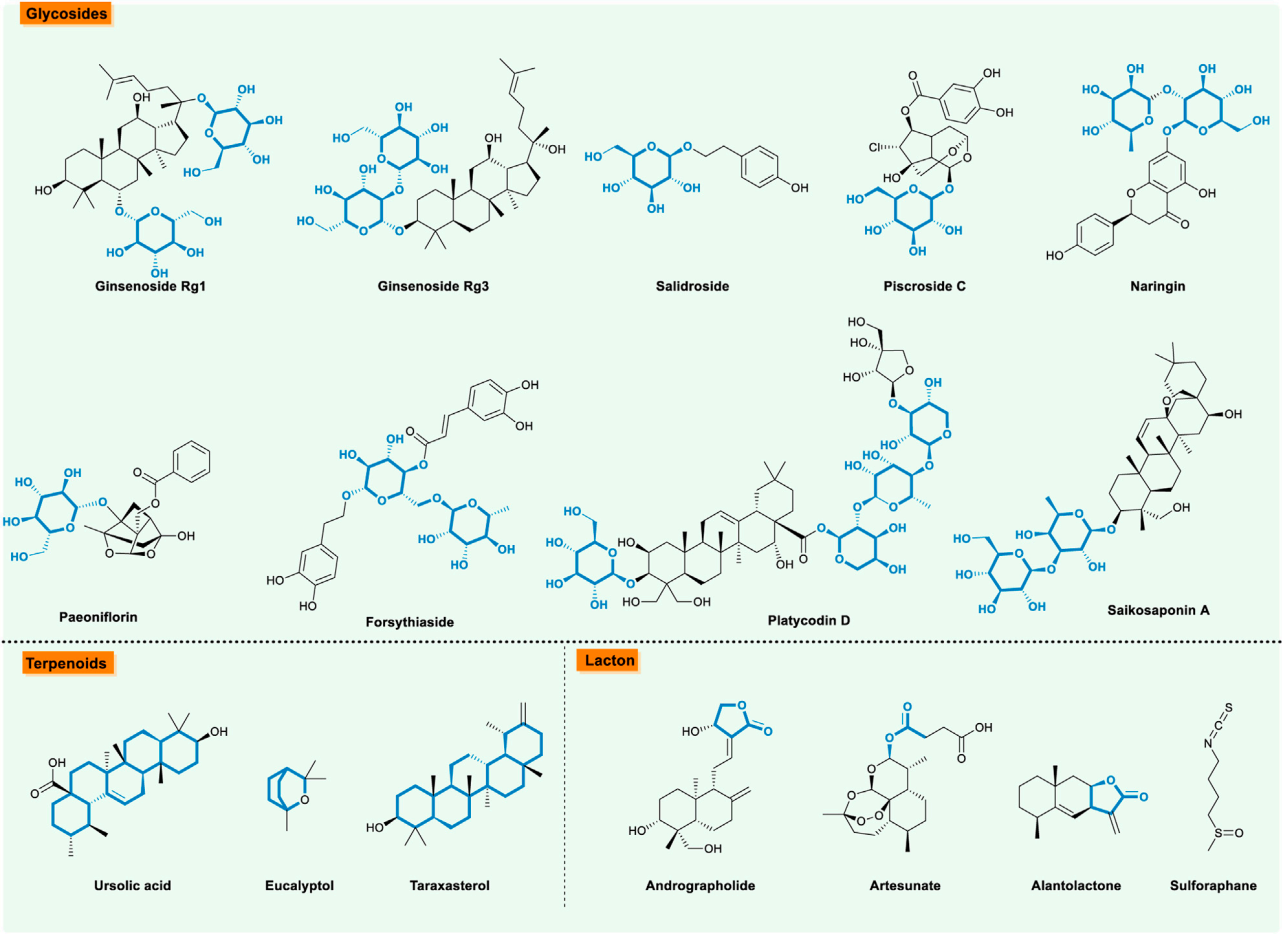
The bioactive natural compounds from glycosides, terpenoids, and lacton.

Glycosides are formed in nature by the interaction of the nucleotide glycosides with the alcoholic or phenolic group, which is categorized as *O-*glycosides, *S*-glycosides, *N-*glycosides, and *C-*glycosides. Among them, this review focuses on *O-*glycosides, the most numerous ones found in nature (Table 4; Figure 5). Ginsenoside Rg1 attenuated CS-induced pulmonary epithelial-mesenchymal transition airway fibrosis by suppressing the TGF-β1/Smad Pathway in both COPD rats and HBE cells (Guan et al., 2017a; Guan et al., 2017b). Subsequently, ginsenoside Rg3 was confirmed that it could suppress neutrophil migration through down-regulating the PI3K pathway, by which ameliorated acute exacerbation of COPD in chronic CS-induced COPD and NTHi-induced acute exacerbation in mice, as well as in BEAS-2B cell models (Guan et al., 2020), which might alleviate acute exacerbation of chronic obstructive pulmonary disease (AECOPD) induced by exacerbation-mediated neutrophilia. Salidroside, one of the extracted compounds of *Rhodiola rosea L.*, was reported to effectively ameliorate an inflammatory response and oxidative stress in COPD model mice induced by CS, which negated the MAPK/NF-kB pathway (Luo et al., 2017). Alongside it, salidroside also mitigated the long-term CS-induced emphysema and skeletal muscle atrophy in rats by inhibiting oxidative stress and inflammatory responses and regulating muscle-specific transcription factor expression (Zhang et al., 2019). Piscroside C, a novel iridoid glycoside isolated from *Pseudolysimachion rotundum* var. *Subinegrum,* was capable of effectively inhibiting inflammatory responses induced by CS, intervening a vital part of COPD development by the way of IKK/NF-κB inhibition (Song et al., 2015). Related research further found that piscroside C inhibited the TNF-α/NF-κB pathway by obstructing the interaction of protein kinase C (PKCδ) towards a TNF receptor 1 signaling complex (TNF-RSC) formation with a model of TNF-α-stimulated human airway epithelial cells (NCI-H292 cells) (Lee SU. et al., 2018). As for naringin, a well-known compound equipped with an effective anti-inflammatory activity, attenuated chronic pulmonary neutrophilic inflammation in CS-exposed rats (Nie et al., 2012). Apart from inflammatory protection, glycosides exhibit diverse biological effects on attenuating COPD progression, for instance, paeoniflorin, a monoterpene glycoside, was reported to re-balance the relationship between oxidant and anti-oxidant in CS-induced mice lung tissues with COPD via a Nrf2-dependent mechanism (Lin et al., 2016). Then, forsythiaside, an active constituent isolated from the Chinese medicinal herb *Forsythia suspensa*, offered protection against CS-induced mice lung injury via activating the Nrf2 and inhibiting the NF-κB signaling pathway (Cheng et al., 2015). Moreover, platycodin D, a major saponin derived from the roots of *Platycodon grandiflflorum*, had been shown to have protection towards CS-induced lung inflammation via suppressing an inflammatory and oxidative response by activating the Nrf2 signaling pathway. This phenomenon indicated that platycodin D might be a promising therapeutic agent for lung inflammation induced by CS (Gao et al., 2017). As for saikosaponin a, a triterpenoid saponin existed in *Radix bupleuri*, was found to ameliorate CS-induced oxidant stress and inflammatory via inhibiting CS-induced NF-κB activation and up-regulating the expression of Nrf2 and HO-1, proving its therapeutic potential towards CS-induced lung inflammation (Chen et al., 2018).

**TABLE 4:** The effects of glycosides on COPD.

Glycosides		Sources	Models	Effects	Dose	Application	Ref
Ginsenoside Rg1	Panax ginseng		CSE-induced COPD mice; Human embryonic lung fibroblasts exposed to CSE	Suppressed airway fibrosis	20 mg/kg	*In vivo*	Guan et al. (2017a)
					40 μM	*In vitro*	
			CSE-induced COPD mice and HBE cells model	Attenuated Pulmonary Epithelial-Mesenchymal Transition (EMT)	5–20 mg/kg	*In vivo*	Guan et al. (2017b)
					5–160 μM	*In vitro*	
Ginsenoside Rg3	Panax ginseng		AECOPD murine model established by CS exposure and NTHi infection; CS- and NTHi stimulation on BEAS-2B	Inhibition of PI3K	10–40 mg/kg	*In vivo*	Guan et al. (2020)
					10–160 μM	*In vitro*	
Salidroside	Rhodiola rosea L		CS-induced COPD in mice	Mitigated skeletal muscle atrophy	50–200 mg/kg	*In vivo*	Zhang et al. (2019)
			CS-induced COPD in mice	Inhibition the MAPK/NF-kB pathway	20–40 mg/kg	*In vivo*	Luo et al. (2017)
Piscroside C	Pseudolysimachion rotundum var. subintegrum		TNF-α-stimulated human airway epithelial cells (NCI-H292 cells)	Inhibited TNF-α/NF-κB pathway by suppression of PKCδ activity for TNF-RSC formation	2.5–20 μM	*In vitro*	Lee et al. (2018b)
			CS- and LPS-induced COPD mice model; TNF-stimulated human airway epithelial NCIH292 cells	Suppression of IKK/NF-κB activation	15–30 mg/kg	*In vivo*	Song et al. (2015)
					2.5–20 μM	*In vitro*	
Naringin	Grape fruit and citrus fruits		CS-induced COPD mice model	Anti-inflammatory	20–80 mg/kg	*In vivo*	Nie et al. (2012)
Paeoniflorin	Paeonia lactiflora		CS-exposed COPD mice model	Attenuated oxidative stress via an Nrf2-dependent mechanism	40 mg/kg	*In vivo*	Lin et al. (2016)
Forsythiaside	Forsythia suspensa		CS-induced mice model	Activating Nrf2 and inhibiting NF-κB signaling pathways	15–60 mg/kg	*In vivo*	Cheng et al. (2015)
Platycodin D	Platycodon grandiflflorum		CS-induced mice model	Activating the Nrf2 signaling pathway	20–80 mg/kg	*In vivo*	Gao et al. (2017)
Saikosaponin a	Radix bupleuri		CS-induced mice model	Inhibited oxidant stress and inflammatory by activating the Nrf2 and inhibiting the NF-κB signaling pathway	5–20 mg/kg	*In vivo*	Chen et al. (2018)

## Terpenoids

Terpenoids represent a highly diverse group of natural products with wide applications. Among these, several molecules exhibited positive effects towards COPD (Table 5; Figure 5). Taking ursolic acid as an example, a pentacyclic triterpenoid compound exists in many plants, and has anti-oxidant/anti-inflammatory activities. Studies pointed out that ursolic acid could effectively attenuate CS-induced mice emphysema (Lin et al., 2017; Lin et al., 2019a; Lin et al., 2019b), which might be fullfiled by the down-regulation of the PERK pathway to attenuate apoptosis, with a combination of up-regulation of Nrf2

**TABLE 5:** The effects of terpenoids on COPD.

Terpenoids		Sources	Models	Effects	Dose	Application	Ref
Ursolic acid	Loquat leaves, glossy privet leaves, forsythia, Prunella vulgaris		CSE treated normal human bronchial epithelial (NHBE) cell model; mice model established by A549 cells in nude mice *in vivo*	Prevented development of lung cancer	10 mg/kg	*In vivo*	Liu et al. (2012)
					3.2–25 μmol/L	*In vitro*	
			CS-induced mice emphysema model	Down-regulating PERK pathway and up-regulating Nrf2 pathway	10–40 mg/kg	*In vivo*	Lin et al. (2017)
			CS-induced emphysema mice	Alleviated airway-vessel remodeling and muscle consumption partly through IGF1 and TGF-β1/Smad2.3 signaling pathways	10–40 mg/kg	*In vivo*	Lin et al. (2019b)
			CES-exposed mice model	Alleviated CSE-induced emphysema and airway remodeling	10–40 mg/kg	*In vivo*	Lin et al. (2017)
Eucalyptol	Eucalyptus globulus		CS-induced COPD mice model	Promoted lung repair	1–10 mg/kg	*In vivo*	Kennedy-Feitosa et al. (2019)
			CS-induced COPD mice model	Anti-inflammatory and antioxidant effects via attenuating NF-κB p65 subunit activation	1–10 mg/ml	*In vivo*	Kennedy-Feitosa et al. (2016)
			CS-induced COPD mice model	Against bacterial invasion through attenuating ciliated cell Damage and suppressing MUC5AC expression	260 mg/kg	*In vivo*	Yu et al. (2019a)
			CS-induced COPD mice model	Mitigated lung injury by suppressing ICAM-1 gene expression	260 mg/kg	*In vivo*	Yu et al. (2018b)
Taraxasterol	Taraxacum officinale		CS-induced mice model; CSE- induced HBE cells model	Inhibiting oxidative stress and inflammatory responses	2.5–10 mg/kg	*In vivo*	Xueshibojie et al. (2016)
					3–12 μg/ml	*In vitro*	

A pathway to modify oxidant stress in CS-induced mice lungs (Lin et al., 2017), following reports from Lin et al. further proved that ursolic acid could regulate IGF1 and TGF-β1/Smad2.3 signaling pathways (Lin et al., 2019b) and three unfolded protein response (UPR) pathways. Notably, ursolic acid could also attenuate downstream apoptotic pathways, as well as the activation of Smad2 and Smad3 (Lin et al., 2019a) regulating. Meanwhile, Wenbo Liu et al. uncovered that ursolic acid was able to inhibit CSE-induced NHBE cell injuries and prevent the development of lung cancer, which indicated that ursolic acid was a promising chemopreventive agent of lung cancer (Liu et al., 2012). As a saturated monoterpene, eucalyptol was reported as an anti-oxidant and anti-inflammatory candidate for the treatment of CS-induced COPD in mice (Kennedy-Feitosa et al., 2016; Yu N. et al., 2018; Kennedy-Feitosa et al., 2019) through the promotion of lung repair. As for the mechanisms of eucalyptol on anti-oxidant and anti-inflammation, preliminary work found that the desirable effects were related to the attenuation of NF-κB p65 subunit activation (Kennedy-Feitosa et al., 2016). Futhermore, Yu et al. indicated that these biological functions conducted by eucalyptol was not only highly associated with the suppression of intercellular adhesion molecule (ICAM)-1 gene expression in diseased lungs (Yu N. et al., 2018), but also with ciliated cell damage attenuation and MUC5AC expression inhibition, thus protecting the lungs from bacterial invasion through a joint mechanism (Yu N. et al., 2019). Finally, taraxasterol, a pentacyclic-triterpene isolated from *Taraxacum officinale,* could effectively work against CS-induced lung inflammation in mice and in HBE cells via inhibiting reactive oxygen species (ROS)-induced TLR4 trafficking to lipid rafts (Xueshibojie et al., 2016).

## Lactone

Lactone, a class of cyclic organic esters, is known as the outstanding exponents of secondary metabolites because of their remarkable biological activities and chemical architectures (Table 6; Figure 5). Regarding the biological functions of lactone upon COPD, four representative nature products are listed below. Firstly, Andrographolide, a labdane diterpene lactone isolated from the *Andrographis paniculata* plant, was reported to be a great candidate for therapy on the CS-induced COPD model *in vivo* and *in vitro* due to its anti-lung inflammation and anti-oxidative stress injury (Guan et al., 2013; Li et al., 2013; Yang et al., 2013; Tan et al., 2018; Zhang et al., 2020) via the complex mechanisms including activation of HO-1 (Yang et al., 2013), inhibition of SIRT1/ERK signaling (Zhang et al., 2020), induction of microRNA-218 (Li et al., 2013), and the augmentation of Nrf2 activity (Guan et al., 2013; Tan et al., 2018). Secondly, artesunate, a semi-synthetic derivative of artemisinin, possessed characteristics of anti-inflammatory and anti-oxidative effects on CS-induced lung impairments by suppressing the PI3K and p42/22 MAPK signaling pathways, enhancing Nrf2 and catalase activities, and reducing the NOX2 level (Ng et al., 2014). Furthermore, Kunming Pan et al. revealed that the artesunate treatment significantly protected against CS-induced airway inflammation, as well as airway remodeling via PPAR-γ/TGF-β1/Smad2/3 signaling pathway *in vivo* and *in vitro* (Pan et al., 2021). Thirdly, the natural sesquiterpene lactone alantolactone (ALT), which was isolated from *Inula helenium L*, possessed the abilities of suppressing CSE-induced inflammation, apoptosis, and oxidative stress in BEAS-2B and NHBE cells via modulating the NF-κB and Nrf2/HO-1 axis (Dang et al., 2020). Lastly, sulforaphane, an isothiocyanate derived from cruciferous vegetables, was famous for its anti-inflammatory activities. Xiaoli Zeng et al. indicated that sulforaphane exerted anti-inflammatory activities in monocyte-derived macrophages (MDMs) from patients with COPD by modulating the toll-like receptors’ (TLRs) pathway, which suggested that sulforaphane may be a potential therapeutic agent for the treatment of COPD (Zeng et al., 2021).

**TABLE 6:** The effects of lactone on COPD.

Lactone			Sources	Models	Effects	Dose	Application	Ref
Andrographolide	Andrographis paniculata			CSE-exposed RAW 264.7 cells	Inhibition of SIRT1/ERK signaling pathway	1–40 µM	*In vitro*	Zhang et al. (2020)
				BEAS-2B cells exposed to CSE	Augmented Nrf2 antioxidant defense and facilitated autophagic flux blockade	10–30 μM	*In vitro*	Tan et al. (2018)
				Human alveolar epithelial A549 cells exposed to CSE	Induction of microRNA-218	5 μM	*In vitro*	Li et al. (2013)
				CSE-exposed bronchial epithelial cells (BEAS-2); CS-exposed mice as COPD model	Augmentation of Nrf2 activity	0.1–1 mg/kg	*In vivo*	Guan et al. (2013)
						30 μM	*In vitro*	
				CS-exposed mice model	Activation of HO-1–mediated signaling	1 mg/kg	*In vivo*	Yang et al. (2013)
Artesunate	Artemisia annua L			CS-exposed COPD mice model; human bronchial smooth muscle cells exposure in CSE	Against airway inflammation and airway remodeling via PPAR-γ/TGF-β1/Smad2/3 signaling	25–100 mg/kg	*In vivo*	Pan et al. (2021)
						1–100 μM	*In vitro*	
				CSE-exposed BEAS-2; CS-exposed mice as COPD model	Anti-inflammatory and anti-oxidative	10–100 mg/kg	*In vivo*	Ng et al. (2014)
						30 μM	*In vitro*	
Alantolactone	Inula helenium L			CSE-exposed BEAS-2B and NHBE cells	Activation of Nrf2/HO-1 and inhibition of the NF-κB pathways	1–10 μM	*In vitro*	Dang et al. (2020)
Sulforaphane	Cruciferous vegetables			Monocyte-derived macrophages (MDMs) from patients with COPD	Modulating the TLR pathway	2.5–20 μmol/L	*In vitro*	Zeng et al. (2021)

## Acid

**FIGURE 6:**
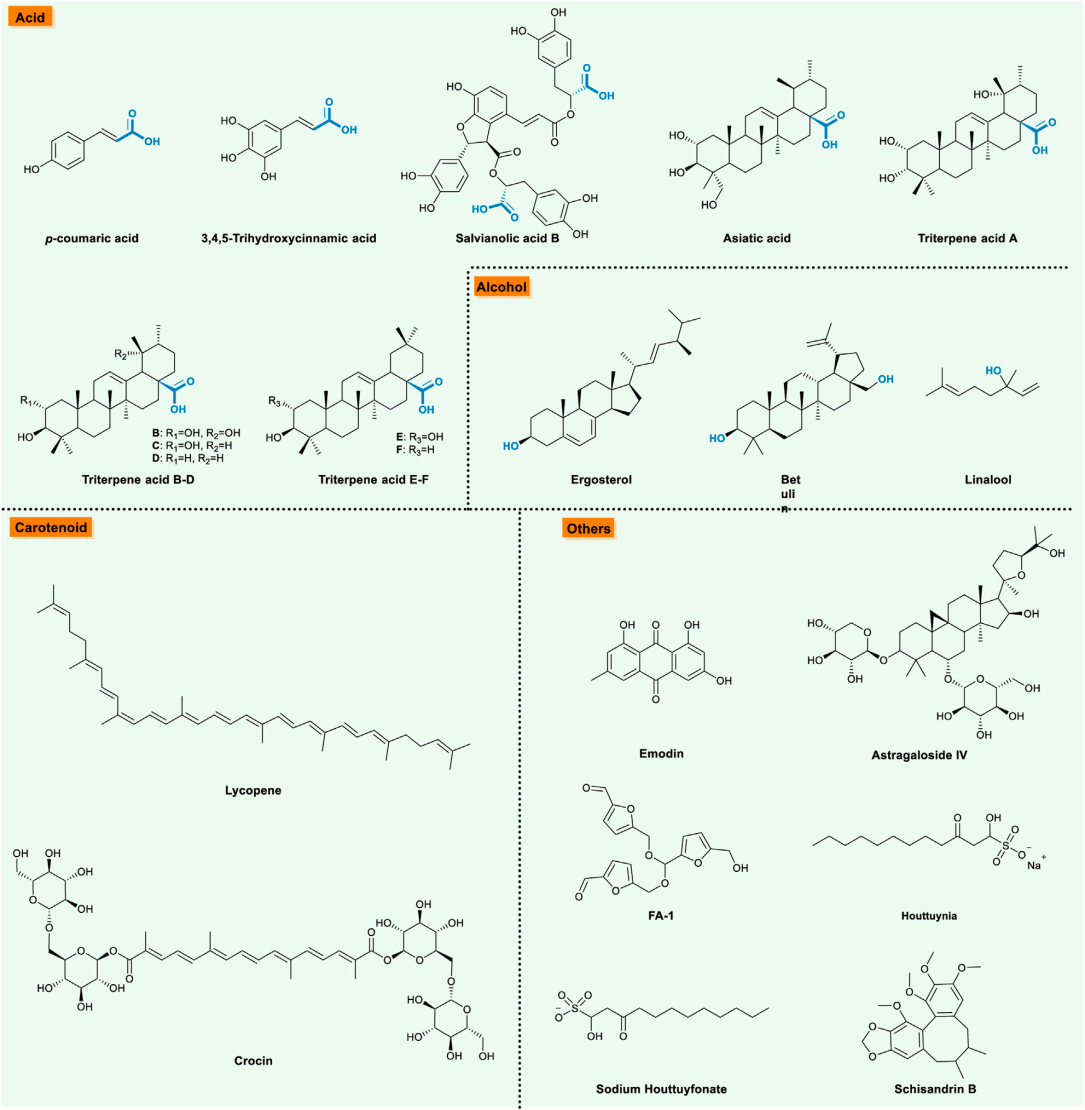
The bioactive natural compounds from acid, alcohol, carotenoid –and others.

Organic acids are classified as compounds bearing carboxylic acid groups from the view of chemistry, which are widely distributed in nature. With regard to COPD, organic acids contribute anti-inflammatory and anti-oxidant effects (Table 7; Figure 6). For instance, *p*-Coumaric acid, a phenolic acid, effectively decreased the production of IL-8 in CSE-stimulated A549 cells as efficiently as dexamethasone, the standard drug for research of the inflammatory process (da Silva et al., 2019). Besides, Woogyeong Kim et al. described that *p*-coumaric acid displayed an anti-inflammatory effect in the CS-induced pulmonary inflammation mice model by inhibiting pro-inflammatory mediators such as cytokines and chemokine, via blocking NF-κB translocation to the nucleus (Kim et al., 2018). (query)3,4,5-trihydroxycinnamic acid, a derivative of hydroxycinnamic acid, ameliorated pulmonary inflammation in mice due to CS exposure and LPS administration by suppressing inflammatory molecules and inflammatory cell recruitment accompanied by suppressing MAPK (partial p38 and JNK) and NF-κB signaling. Notably, 3,4,5-trihydroxycinnamic acid pre-treatment reduced PMA-triggered IL-6 secretion in A549 or H292 cells by up-regulating NAD(P)H dehydrogenase (quinone 1) 1 (NQO1) expression (Min et al., 2020). Moreover, salvianolic acid B, a useful compound isolated from the Chinese herb *Radix salviae Miltiorrhizae,* exhibited both anti-oxidant and anti-inflammatory effects against CS-induced lung inflammation via activating Nrf-2 and inhibiting NF-κB activation, which suggested that salvianolic acid B treatment may be a potential therapy option while treating COPD (Zhang DF. et al., 2015). In addition, asiatic acid is one of the major components of the titrated extract of *Centella asiatica* (TECA), could effectively protect against pulmonary inflammation and mucus overproduction by inhibition of inflammatory molecules via suppressing the activation of MAPKs and NF-κB pathway, up-regulating HO-1 in the lung tissue of CS exposure mice at the meantime (Lee et al., 2016). As a series of bioactive acids extracted from loquat leaves, triterpene acids suppressed the production of inflammatory mediators on CS-induced COPD mice in a dose-dependent manner via modulating CS-induced AMPK/Nrf2 and NF-κB/iNOS signaling pathways (Jian et al., 2020).

**TABLE 7:** The effects of acid on COPD.

Acid	Sources	Models	Effects	Dose	Application	Ref
p-coumaric acid	Bambusae Caulis	A549 cells exposed to CSE to induce inflammatory process	Anti-inflammatory	10–100 µM	*In vitro*	da Silva et al. (2019)
		CS-induced inflammatory mice model	Suppressed CS-induced pulmonary inflammation	5–10 mg/kg	*In vivo*	Kim et al. (2018)
3,4,5-Trihydroxycinnamic acid
	Cinnamomum cassia Presl	COPD model elicited by CS and LPS; phorbol myristate acetate (PMA)-stimulated A549 or H292 airway epithelial cells	Down-regulation of MAPK (partial p38 and JNK)/NF-κB signaling and upregulation of NQO1 and SIRT1 expression	20–40 mg/kg	*In vivo*	Min et al. (2020)
				5–50 µM	*In vitro*	
Salvianolic acid B	Radix Salviae Miltiorrhizae	CS-induced mice model	Attenuated inflammation via activating Nrf-2 and inhibiting NF-κB activation	6–25 mg/kg	*In vivo*	Zhang et al. (2015a)
Asiatic acid	Centella asiatica	CS-exposed mice model	Up-regulation of HO-1 and inhibition of the activation of MAPKs and NF-kB pathway	15–30 mg/kg	*In vivo*	Lee et al. (2016)
Triterpene acids	Eriobotrya japonica	CS-induced mice model	Regulating the AMPK/Nrf2 and NFκB Pathways	50–100 mg/kg	*In vivo*	Jian et al. (2020)

## Alcohol

Alcohol, a class of organic compounds characterized by one or more hydroxyl (―OH) groups attached to a carbon atom of an alkyl group. Among numerous alcohols exist in nature, several compounds exhibit therapeutic effects on the COPD model (Table 8; Figure 6). Citing ergosterol for instance, the main bioactive ingredient in *Cordyceps sinensis* (*C. sinensis*), suppressed COPD inflammatory, oxidative stress, and apoptosis in both CSE-induced 16HBE cells and Balb/c mice via inhibiting the activation of NF-κB/p65, suggesting that ergosterol may be partially responsible for the therapeutic effects on COPD patients (Sun et al., 2019). More evidences, like Wang Huan et al., demonstrated the protective effects of ergosterol on CS-induced COPD mice manifesting as an anti-inflammatory response possibly by inhibiting the JAK3/STAT3/NF-κB pathway (Huan et al., 2017). As for betulin, a pentacyclic triterpene alcohol, which is extracted from the bark of the birch tree, was reported to show protective effects on CS-induced COPD mice by inhibiting inflammatory response and oxidative stress via inhibiting the ROCK/NF-kB pathway (Chunhua et al., 2017). Apart from the two mentioned previously, Linalool, a natural compound existing in the volatile oil of several aromatic plant species, dramatically alleviated CS-induced lung inflammation due to the inhibition the inflammatory cell infiltration and TNF-α, IL-6, IL-1β, and IL-8 production by inhibiting CS-induced NF-κB activation in a dose-dependent manner (Ma et al., 2015).

**TABLE 8:** The effects of alcohol on COPD.

Alcohol		Sources	Models	Effects	Dose	Application	Ref
Ergosterol	Cordyceps sinensis (C. sinensis)		CSE-induced COPD model both in 16HBE cells and Balb/c mice	Suppressed COPD inflammatory and oxidative stress and apoptosis through the suppression of NF-κB/p65 activation	20–40 mg/kg	*In vivo*	Sun et al. (2019)
					5–20 μM	*In vitro*	
			CS-induced COPD mice model	Inhibiting the JAK3/STAT3/NF-κB pathway	25–50 mg/kg	*In vivo*	Huan et al. (2017)
Betulin	Birch tree bark		CS-induced COPD mice model	Inhibiting the inflammatory response and oxidative stress possibly through the ROCK/NF-κB pathway	20–40 mg/kg	*In vivo*	Chunhua et al. (2017)
Linalool	Aromatic plants species		CS-induced COPD mice model	Against inflammation by inhibiting CS-induced NF-κB activation	10–40 mg/kg	*In vivo*	Ma et al. (2015)

## Carotenoid

Carotenoids are lipid-soluble pigments and naturally exist in flora and fauna, which offer multiple beneficial functions (Table 9; Figure 6). With regard to the theme this review focuses on, lycopene, a carotenoid found in plant foods, was found to demonstrate anti-oxidant and anti-inflammatory properties in mice exposed to long/short-term CS exposure (Campos et al., 2017; Campos et al., 2019). Overall, the consumption of lycopene in the diet might contribute to the prevention of and therapy for treatment of patients with COPD. Besides, crocin, a valuable constituent of *Crocus sativus* L, effectively against CS-induced COPD complicated with comorbid depression, due to its inhibition of the inflammatory response via PI3K/Akt-mediated NF-κB signaling (Xie et al., 2019). For another, Mahin Dianat et al. found that crocin could protect the lungs against injuries and related cardiac dysfunction caused by COPD via modulation of the Nrf2 pathway among CS exposure mice models. (Dianat et al., 2018).

**TABLE 9:** The effects of carotenoids on COPD.

Carotenoid		Sources	Models	Effects	Dose	Application	Ref
Lycopene	Tomatoes		CS-exposed mice model	Anti-oxidant and anti-inflammatory	25–50 mg/kg	*In vivo*	Campos et al. (2019)
			J774A.1 (Macrophages) cells exposed to CSE; CS-exposed mice model	Anti-oxidant and anti-inflammatory	25–50 mg/kg	*In vivo*	Campos et al. (2017)
					0.5–25 µM	*In vitro*	
Crocin	Crocus sativus L		CS-induced mice model	Activation of Nrf2 pathway	50 mg/kg	*In vivo*	Dianat et al. (2018)
			CS-exposed C57BL/6 mice model	Preventing the activation of PI3K/Akt mediated NF-κB inflammatory pathways	50 mg/kg	*In vivo*	Xie et al. (2019)

## Others

Apart from the valuable natural compounds previously summarized, there are other numerous natural products with different scaffolds that contribute therapeutic functions towards COPD (Table 10; Figure 6). For instance, emodin, an active compound of *Rheum palmatum* L., demonstrated protective effects against lung inflammation and oxidative injury induced by CS in mice model via enhancing the expression and activities of HO-1 and Nrf-2 (Xue et al., 2015). Alongside it, astragaloside IV, the best biological activity among *Astragalus polysaccharide,* could provide protection both on CS-induced COPD in mice and in human bronchial epithelial cell models via blocking the JAK3/STAT3/NF-κB pathway (Meiqian et al., 2018). Meanwhile, polysaccharides from *Dendrobium huoshanense* stems alleviated CS-induced lung inflammation in mice via inhibiting the NF-κB and MAPK signaling pathways (Ge et al., 2018), while 5,5'-((((5-(hydroxymethyl)furan-2-yl)methylene)bis (oxy))bis (methylene))bis (furan-2-carbaldehyde) (FA-1) isolated from a concentrated Japanese apricot extract (JAE), enabled protection against cytotoxicity, DNA damage, and oxidative stress in CSE-exposed HBE cells and normal human epidermal keratinocyte (NHEK) cells via augmenting aldehyde dehydrogenase (ALDH) and DNA repair (Jang et al., 2018). Furthermore, houttuynia, one of the main components of the cordate houttuynia, could alleviate lung injury in the rats’ lung tissues of COPD induced by smoking combined with intratracheal instillation of LPS via inhibiting the TLR4/MyD88/NF-κB activation sequence (Wang et al., 2021). As a bioactive compound extracted from houttuynia, sodium houttuyfonate (SH) significantly alleviated the pulmonary inflammation via suppressing the TLR4/NF-κB pathway, thus protecting the lung tissue on the CS-/LPS-induced mice model with COPD (Wu et al., 2017), and schisandrin B, a dibenzocyclooctadiene derivative identified from *Schisandra chinensis*, was reported to fight against CS-induced lung inflammation in mice by activating the Nrf2 and inhibiting NF-κB signaling pathway (Jia et al., 2017).

**TABLE 10:** The effects of other compounds on COPD.

Compound	Sources	Models	Effects	Dose	Application	Ref
Emodin	Rheum palmatum L	CS-induced lung injury in a mouse model	Enhancing the expression and activities of HO-1 and Nrf-2	20–40 mg/kg	*In vivo*	Xue et al. (2015)
Astragaloside IV	Astragalus mongholicus	CS-induced mice model; CSE-stimulated NHBE cells model	Inhibition of the JAK3/STAT3/NF-κB pathway	10–40 mg/kg	*In vivo*	Meiqian et al. (2018)
				10–40 μM	*In vitro*	
Polysaccharides from Dendrobium huoshanense	Dendrobium huoshanense	CS-induced mice model	Inhibition of the NF-κB and MAPK signaling pathways	100–400 mg/kg	*In vivo*	Ge et al. (2018)
FA-1	Prunus mume	CSE-induced immortalized HBE cells and normal human epidermal keratinocytes (NHEK)	Augmenting ALDH and DNA repair	150 nM	*In vitro*	Jang et al. (2018)
Houttuynia	Houttuynia cordata Thunb	Mice model of COPD established by smoking combined with intratracheal instillation of LPS	Inhibiting the activation of the TLR4/MyD88/NF-κB (p65) signaling pathway	5–25 mg/kg	*In vivo*	Wang et al. (2021)
Sodium Houttuyfonate	Houttuynia cordata Thunb	CS- and LPS-induced mice model	Suppressing the TLR4/NF-κB pathway	24.3 mg/kg	*In vivo*	Wu et al. (2017)
Schisandrin B	Schisandra chinensis	CS-induced mice model	Activating Nrf2 and inhibiting the NF-κB signaling pathway	20–80 mg/kg	*In vivo*	Jia et al. (2017)

## Summary

This review discloses that LPS, cigarette smoke, and cigarette smoke extract contribute to the development of COPD, and the cellular biological processes concerning COPD mainly involve immune inflammatory response, apoptosis, fibrosis, and oxidative stress, which gradually lead to airway structural changes, obstruction, and destruction of the alveolar structure and respiratory symptoms. Moreover, these reported natural small molecular compounds demonstrated unique functions in the treatment of COPD through numerous biological processes such as anti-inflammatory, anti-oxidant, anti-apoptosis, and anti-airway fibrosis, as shown in Figure 2. The main signaling pathways involved in the regulation of physiological functions of lung cell or tissue refer to the JAK3/STAT3/NF-κB and MAPK inflammatory signaling pathways, the Nrf2 oxidative stress signaling pathway, TGF-β1/Smad 2/3 fibrosis signaling, and so on; related targets are mainly about TNF-α, IL-6, IL-8, TIMP-1, MMP, AKT, JAK3, IKK, PI3K, HO-1, MAPK, P38, ERK, etc. as shown in Figure 3. It is worth noting that a few compounds (like baicalin, quercetin, resveratrol, curcumin, and ursolic acid) have shown impressive effects on improving COPD symptoms, considering the great potential of these valuable molecules, continuous efforts should be paid in this field, especially from a simple molecular level to a mechanism level. Besides, the efficacy of the single-drug curative strategy is far from the clinical needs in the current CODP treatment, and this inspires researchers that a combination strategy utilizing two or more bioactive natural compounds seems to be a potential direction of COPD research (Terry and Dhand, 2020). Not only could this therapeutic combination increase the degree of bronchiectasis, but also reduce the toxic and side effects by reducing the dosage and enhancing complementary therapeutic effects of the bioactive molecule used. In brief, natural small molecular compounds demonstrate great potential in the area of COPD treatment, and we hope that this review can bring a quick look and provide some inspiration for the research in relevant fields.
